# Supplementary material for: Correlates of behavioral and emotional disorders among school-going adolescents in Uganda
Source: PLOS Glob Public Health. 2025 Jun 12;5(6):e0003907. doi: 10.1371/journal.pgph.0003907 (PMC12161558; doi:10.1371/journal.pgph.0003907)
Supplement: Attachment — Submitted filename: Comments by Tamene Berhanu december 28, 2024.docx [file pgph.0003907.s004.docx]

**Date 9/15/2024**

PLOS Global Public Health

Correlates of behavioral and emotional disorders among school-going adolescents in Uganda

--Manuscript Draft—

**Manuscript Number: PGPH-D-24-02392**

**Reviewed by Tamene Berhanu Alaho (**Mental Health Specialist**)**

**Comments.**

1. **Background**

It is better to put statement in paragraph two in the place of paragraph one; please revise this section to make clear understanding for your study.

2. **Method**

Who are respondents for this study? - Adolescents or their parents or both of them. Please make it clear and describe in details to reduce bias. Collecting data from adolescents or from their parents raise the risk of recall bias; so you should be explain what you did to reduce information bias from respondents in limitation section of your study.

3. **Conclusion**

Recommendation should be based on finding, you can recommend for factors associated with outcome variable in your study. Make it smart since conclusion is final output for your study.

Final what you did for the respondents who had emotional and behavioral problems in your study. Please specify your intervention for them if did for them.

4. Figures not visible, revise it
5. Check the references for clearly state author, journal name, volume, page number, etc.
