# Supplementary material for: Correlates of behavioral and emotional disorders among school-going adolescents in Uganda
Source: PLOS Glob Public Health. 2025 Jun 12;5(6):e0003907. doi: 10.1371/journal.pgph.0003907 (PMC12161558; doi:10.1371/journal.pgph.0003907)
Supplement: Attachment — Submitted filename: Response to Reviewers.docx [file pgph.0003907.s006.docx]

**Date 9/15/2024**

PLOS Global Public Health

Correlates of behavioral and emotional disorders among school-going adolescents in Uganda

--Manuscript Draft—

**NOTE: All callouts to specific line numbers or page numbers correspond to the file titled “Revised Manuscript with Tracked Changes”.**

**Manuscript Number: PGPH-D-24-02392**

**Reviewer #1:** This is an interesting study on cross-sectional associations between demographic, school characteristics, and hardship, and mental health of adolescents in Uganda. A methodic and efficient approach was used for the sampling during 6 months of 2017, which is well-described in the methods. The sample size seems adequate for the study. The overall aim of the study is to identify at-risk individuals in order to inform intervention strategies for youth mental health in the school setting.

I have a few general comments: I recommend reviewing the structure of the paragraphs to make sure they are linked to one another, using transitions. Most importantly, I recommend a more expansive literature review for both the background and the discussion. More context in which the present study places itself is needed, and more interpretation of the findings in light of prior studies in Uganda or Sub-Saharan Africa is necessary for a more impactful discussion.

Please find more detailed comments per section.

Background

Please add a reference for the second sentence stating a prevalence of 13.9% of mental health disorders in the global population.

**Response: We added the reference in line 88, Page 2.**

Add a reference for the third sentence stating a 13% burden of disease in adolescents.
**Response: We added the reference in line 89, Page 2.**

For the sentence “Mental disorders (…) have risen considerably”, can you provide an idea of the time frame in which this rise has occurred. Also, the reference for this statement is a study from Uganda. It’s unclear whether the statement focusing on LMICs is adequately supported by this reference.

**Response: We have added the timeframe (since 1990) and adjusted the citation for this sentence on Page 1 (Lines 89-91).**

Reference required to support the statement “Depression, anxiety, and behavioral disorders are among (…)”

**Response: We added a reference in line 93, Page 2.**

There is an incoherence in the background from the abstract and the background in the paper. The abstract reports 11.5% of Ugandan adolescents report emotional disorders (which include depression), vs the background in the manuscript reports 23.6% depression prevalence in Ugandan children and adolescents. The abstract does not specify that 11.5% refers to an at-risk population: perinatally HIV-infected youth.

**Response: We have adjusted the Abstract to not describe individual prevalence values for emotional and behavioral disorders. These values were extracted from a previously used citation which has since been cited differently. There should not be any incoherence anymore.**

It is not clear what the 5th paragraph transition word “therefore” is linked to. It does not appear to fit with the content of the 4th paragraph, but rather the 3rd one. Please review the structure.

**Response: This word has been removed and the syntax has been appropriately adjusted (Line 126 on Page 2)**

The statement “heterogenous methodologies and outcome assessments complicate this field of study” does not appear to be connected to other points made in the paragraph. I suggest removing or clarifying.
“burden” of emotional and behavioral disorders is used multiple times in the background, could you specify what you mean by “burden”, is it the prevalence of these disorders?

**Response: We have removed the suggested section (began on Line 127 on Page 1) and specified what is meant by “burden” (prevalence; Line 105 on Page 2)**

Hardship is a variable of interest, but has not been defined. Could you specify what hardship experiences are included when introducing your study in the last paragraph: “We hypothesized that hardship experiences, demographic characteristics, and school features contribute to and could be associated with the prevalence of emotional and behavioral disorders among school-going adolescents in Uganda.” Same comment for school features, and demographic characteristics.

**Response: We have defined the hardship experiences considered within this study in the suggested section. The addition now lies from Lines 142-143 on Page 3.**

Methods

The abstract background information does not match the methods information on age range. The abstract states participants are aged 10-24 years. The methods state the participants are aged 10-18 years. I assume it’s the latter. Please correct accordingly.

**Response: We have adjusted the abstract accordingly based on Line 152 of the Methods section on Page 3. The correct age range is 10-18.**

Results

Participant demographics

The second sentence states: “An overall proportion of 17.36% (n=340) reported symptoms suggestive of a mental health condition, and 5.71% (n=112) had behavioral disorders”. I suppose “mental health condition” refers to emotional disorders. Please correct accordingly.

**Response: That is correct. It has been adjusted to be emotional disorder in Line 229 on Page 4.**

The sentence “Few respondents (1.48%; n=28) reported living in hut-style, grass-thatched homes, 82.40% (n=1,559) lived in permanent residences, and 16.12% of respondents (n=305) lived in semi-permanent homes (Table 1).” This is the first mention of “grass-thatched homes” which I assume is semi-permanent housing. I suggest to either state earlier that semi-permanent includes grass-thatched homes, or remove grass-thatched in this sentence and replace by “semi-permanent.

**Response: The use of “grass-thatched homes” was meant to clarify what hut-style housing meant. Because this was not clear, we decided to remove mention of the word in Line 235 on Page 4. We will let hut-style home stand alone as a representation of non-permanent housing.**

Prevalence of behavioral disorders

Prevalence for conduct disorders and oppositional disorders are stated, except for ADHD. I suggest adding the prevalence for all behavioral disorders.

**Response: The prevalence of ADHD has been added in Lines 279 to 280 on Page 4.**

This section includes prevalence of emotional disorders, please modify the structure for the reported results to match your sections. Please merge this section with “prevalence and correlates of behavioral disorders”

**Response: We have combined these sections to become “Prevalence and correlates of behavioral disorders” to follow a consistent structure in the Results section. The adjusted section begins on Line 276 on Page 5. We have also added details on the prevalence of emotional disorders in the study population; this section stretches from Line 239 to 274 on Page 4-5.**

Prevalence and correlates of emotional disorders

Define prevalence ratio then use PR subsequently.

**Response: We have written out the acronyms PR and OR in full at first uses in line 220 in Page 4.**

Can you comment on the overlap between emotional and behavioral disorders, did you find participants with both, or are your categories “emotional disorder” and “behavioral disorder” exclusive categories?

**Response: Overall, a small portion of participants (n=55, 2.78%) had both an emotional and behavioral disorder. Of those with an emotional disorder, only about 15% also had a behavioral disorder. We have added a statement on this overlap to Line 230 on Page 4.**

Is Figure 1 just an illustration of your results in Table 2, adjusted models? If so, please state.

**Response: We have added a statement on this to the last sentence of the “Prevalence and correlates of behavioral disorders” section on Page 5. The addition stretches from Lines 289 to 291.**

Please state for what models the logistic vs modified poisson regressions were used in Table 2.

**Response: A statement has been added to the sentence stretching from Lines 289 to 291 in the “Prevalence and correlates of behavioral disorders” section.**

Discussion

Please remove “PTSD” in the second paragraph, it was not included in this study.

**Response: PTSD has been replaced with ADHD (Line 303 on Page 5), which was the intended disorder.**

In the second paragraph, clearly state the prevalence rates in this study compared to the rates reported in other studies, so we understand the differences in prevalence.

**Response: The specific prevalence values have been added to the second paragraph of the Discussion section (Page 5)**

Same comment for the third paragraph, report prevalence rates when stating differences between studies, within country or between countries.

**Response: We have added the specific prevalence values we refer to from the cited studies to the third paragraph of the Discussion section (Page 5).**

It is unclear why India is mentioned, because it is also LMIC?

**Response: Yes, that is correct. We have specified that India is being mentioned because it is another LMIC, like Uganda (see Line 312 on Page 5).**

Could you elaborate on/provide some interpretation on the discrepancy in depression rates in this study and rates observed in the review on Sub-Saharan Africa?

**Response: We include a discussion of the discrepancy in depression outcomes between our study population and a general population in SSA in Lines 280-284 on Pages 5-6 and 445-448 on Page 7. We confirmed depression rates in the study population using two methods (as described within the manuscript), and both methods resulted in minimal depression outcomes. While it is possible that response fatigue and/or survey design may have contributed to this discrepancy, we believe that we have extensively investigated this topic.**

“By using an alternative approach to categorizing (…)” seems to refer to a supplementary analysis done by the authors but has not been previously described. Could this be integrated earlier in the paper?

**Response: We have added a statement in the Study measures sub-section of the Methods section stating that this alternative approach was used to confirm our results (see Lines 189-190 on Page 3). The alternative method used to confirm our study’s findings has been used in a recently published article (see citation #33).**

Could you expand on why emotional disorders may be higher in private schools? Have there been other studies in Uganda linking private school to stress, or mental health, compared to public schools?

**Response: We have described this potential linkage on Page 6 (Lines 378-383). To the authors' knowledge (and through extensive literature searching), there does not currently exist a published study comparing mental health within private and public schools in Sub-Saharan Africa, particularly in Uganda.**

Please include a discussion on the association between family history of mental illness and behavioral disorders.

**Response: We have added a paragraph discussing this association in more depth to Page 6 (Lines 385-390).**

Please include a discussion on variables which were not associated to emotional and behavioral disorders (e.g. domestic violence, orphan, urbanity, nature of housing).

**Response: We have added a paragraph discussing this association in more depth to Page 6 (Lines 392-398).**

Could you give concrete recommendations for interventions in the school-setting, according to prior literature? For example, there is literature associating school violence to mental health in Uganda, specifically in girls (Devries, K. M., Child, J. C., Allen, E., Walakira, E., Parkes, J., & Naker, D. (2014). School violence, mental health, and educational performance in Uganda. Pediatrics, 133(1), e129-e137). Are there other factors which may explain the associations that you find?

**Response: We have added a description of specific interventions that could be implemented in a school-setting to improve mental health of the school’s attendees (Lines 412-433 on Page 6). We cited a 2023 systematic review that found a variety of school-based interventions being studied, many of which were implemented by non-specialists.**

**Reviewer #2: Comments.**

1.Background
It is better to put statement in paragraph two in the place of paragraph one; please revise this section to make clear understanding for your study.

**Response: We have placed the contents of paragraph two at the end of paragraph one as suggested.**

2.Method
Who are respondents for this study? - Adolescents or their parents or both of them. Please make it clear and describe in details to reduce bias. Collecting data from adolescents or from their parents raise the risk of recall bias; so you should be explain what you did to reduce information bias from respondents in limitation section of your study.

**Response: We have specified that responses were self-reported by the adolescents themselves (see Line 170 on Page 3). The CASI-5 battery does not ask participants to reflect back on a specific time frame, but asks participants to respond based on their “general behavior”.**

3.Conclusion
Recommendation should be based on finding, you can recommend for factors associated with outcome variable in your study. Make it smart since conclusion is final output for your study.

**Response: We believe that based on revisions to this manuscript, we have provided adequate descriptions of the findings and their relevance, making the recommendations and suggestions described in the Conclusions section appropriate.**

Final what you did for the respondents who had emotional and behavioral problems in your study. Please specify your intervention for them if did for them.

**Response: In Uganda, Mental Health Services is integrated into primary health care(ref). The respondents who had emotional and behavioural problems where referred to the nearest health facility for further evaluation and management including further referrals to regional referral hospitals in the region of the study. We have added this statement to Lines 195-196 on Page 4.**

4.Figures not visible, revise it

**Response: Figure 1 should have been visible as it was appropriately uploaded to the submission portal. The other reviewer of this paper did not report any issue with viewing our Figure.**

5.Check the references for clearly state author, journal name, volume, page number, etc.

**Response: We have confirmed the accuracy and clarity of all references.**
