# Supplementary material for: Correlates of behavioral and emotional disorders among school-going adolescents in Uganda
Source: PLOS Glob Public Health. 2025 Jun 12;5(6):e0003907. doi: 10.1371/journal.pgph.0003907 (PMC12161558; doi:10.1371/journal.pgph.0003907)
Supplement: Attachment — Submitted filename: Response_to_Reviewers_auresp_2.docx [file pgph.0003907.s007.docx]

PGPH-D-24-02392R1
Correlates of behavioral and emotional disorders among school-going adolescents in Uganda
PLOS Global Public Health

Reviewers' comments:

Reviewer's Responses to Questions

Comments to the Author

6. Review Comments to the Author

Please use the space provided to explain your answers to the questions above. You may also include additional comments for the author, including concerns about dual publication, research ethics, or publication ethics. (Please upload your review as an attachment if it exceeds 20,000 characters)

Reviewer #1: I thank the authors for their responses on the comments of the first round of revisions. However, some issues particularly related to the discussion remain.

**Response:** **The authors would like to thank Reviewer #1 for their thorough comments and revision requests.**

INTRODUCTION

The 4th paragraph of the introduction is not connected to the previous or the next paragraphs (3rd and 5th). Although the content of the paragraph is important because it introduces the outcome measure for this study, which is reported to be validated in Uganda, the relevance of the content is not stated. I strongly suggest adding transition sentences between paragraphs in general. The flow of ideas (how each paragraph leads to the next) is not clear.

**Response: Transition sentences have been added between lines 106 and 111.**

RESULTS

Reiterating my previous comment: results on emotional disorders are presented in the behavioral disorders section of your results.

**Response: The few sentences that were discussing depression (an emotional disorder) in the behavioral disorder section have been moved appropriately. They now lie from lines 218-222. We have also added a citation to this section because the alternative analysis we described has since been published since the last round of revisions. It is listed as Reference #30.**

DISCUSSION

In the discussion, you list emotional disorders "The prevalence of emotional disorders (depression, generalized anxiety, ADHD, social anxiety, and separation anxiety)", however ADHD is in your "behavioral disorders" in your results section. Remove ADHD.

**Response: ADHD has been removed from Line 255.**

Please add a hypothesis as to why your study's depression prevalence rates are so low (0.4%) compared to other samples. In your response you mention response fatigue, survey design; how would this contribute to reporting low depressive symptoms? Can you add a hypothesis in your discussion as to why your rates may be so low.

**Response: We have added a few hypotheses from Lines 286-291.**

In the paragraph on family history of mental health predicting behavioral disorders, please expand on why this association was found, but not with emotional disorders. You mention how family dynamics may play a role in youth mental health, expand on how that is linked to mental illness in the family and youth mental health, specifically behavioral disorders.

**Response: An expansion of this section is provided in Lines 311-316.**
